# Continuous positive airway pressure therapy reduces the levels of catecholamines and blood pressure in pseudophaeochromocytoma with coexisting obstructive sleep apnoea

**DOI:** 10.1177/2048004021992191

**Published:** 2021-03-17

**Authors:** Gie Ken-Dror, Michael Wood, David Fluck, Pankaj Sharma, Christopher H Fry, Thang S Han

**Affiliations:** 1 Institute of Cardiovascular Research, Royal Holloway, University of London, Egham, Surrey, UK; 2 Department of Respiratory Medicine, Ashford and St Peter’s Hospitals NHS Foundation Trust, Guildford Road, Chertsey, Surrey, UK; 3 Department of Cardiology, Ashford and St Peter’s Hospitals NHS Foundation Trust, Guildford Road, Chertsey, Surrey, UK; 4 School of Physiology, Pharmacology and Neuroscience, University of Bristol, Bristol, UK; 5 Department of Endocrinology, Ashford and St Peter’s Hospitals NHS Foundation Trust, Guildford Road, Chertsey, Surrey, UK

**Keywords:** Hypertension, obesity, stress hormones, sympathetic activity

## Abstract

**Background:**

Stress from obstructive sleep apnoea (OSA) stimulates catecholamine release and consequently can exacerbate hypertension, even in the absence of a catecholamine-producing tumour (phaeochromocytoma). As such, a positive screening test for suspected phaeochromocytoma may be misleading. There exists only a handful case reports, and no controlled trials, how continuous positive airway pressure (CPAP) to treat OSA influences catecholamine levels. We examined changes to levels of urinary catecholamine and blood pressure in response to CPAP treatment.

**Methods:**

We conducted a meta-analysis of data aggregated from published case reports of individual patient data up to April 2020. The quality of the reports was evaluated using the risk of bias in non-randomized studies of interventions (ROBINS-I) tool.

**Results:**

A total of 13 cases (seven men and six women) from seven reports met our search criteria. Patients had mean age of 49.1 years (range = 36–62) and body mass index of 37.4 kg/m^2^ (range = 27–56). Most had moderate to severe OSA with CPAP treatment. Nine cases had 24-hour urinary noradrenaline assessment before and after CPAP treatment. CPAP treatment led to a 21% reduction (104 nmol/24-hours, 95% credible interval =59 to 148) in 24-hour urinary noradrenaline to within reference ranges, and 25% reduction (from 131 to 100 mmHg) in mean arterial pressure. The risk of overall bias evaluated by the ROBINS-I tool was found to be low in the majority of reports.

**Conclusions:**

Investigations of patients suspected of phaeochromocytoma, particularly obese individuals, should exclude OSA and treat this condition if present before performing screening tests to assess for catecholamine levels.

## Introduction

In normal physiological states, catecholamines are rapidly released as an acute response to stress through activation of the sympathetic system and they return to baseline levels once the stress is withdrawn.^1^ In contrast, patients with phaeochromocytoma, whereby excessive catecholamines are episodically released into the circulation by a tumour, can present with paroxysmal or persistent hypertension and a number of symptoms such as headaches, palpitations and sweating.^2^ In clinical practice, when phaeochromocytoma is suspected, the initial endocrine investigation frequently involves screening tests such as 24-hour urinary free catecholamines: dopamine, adrenaline and noradrenaline, or their products metadrenalines (metanephrines) 3-methoxytyramine, normetadrenaline (normetanephrine) and metadrenaline (metanephrine).^2,3^

Obstructive sleep apnoea (OSA) commonly occurs among individuals with essential hypertension, ranging between 30 and 50% of cases,^4–6^ but many remain undiagnosed.^7,8^ OSA elicits significant stress to the patient, stimulating the release of catecholamines and as a consequence can exacerbate hypertension, even in the absence of a phaechromocytoma.^9,10^ In such patients, a positive screening test may therefore falsely indicate the presence of a phaeochromocytoma. Hitherto, only a handful of case reports have been published showing reductions in the levels of catecholamines or metanephrines by treatment with continuous positive airway pressure (CPAP). Therefore, due to a lack of randomised controlled trials, results from existing case reports are not well publicised or included in clinical guidelines for investigation of phaeochromocytoma.^11^ Consequently, many patients are subjected to unnecessary investigations including radiological procedures and complex endocrine dynamic function tests. These investigations require hospital visits and admissions which may cause anxiety to the patient and impose a substantial cost to healthcare services.

This study was undertaken to determine whether OSA should be considered in cases where there is some indication that a phaeochromocytoma is present, before further evaluation, by conducting a meta-analysis of data aggregated from published clinical case reports of pseudophaeochromocytoma.

## Methods

### Search criteria

Two investigators performed independently a literature search of MEDLINE and Google Scholar up to April 2020 using the key terms (British or US usage and abbreviations, *e.g.* CPAP and OSA): obstructive sleep apnoea, continuous positive airway pressure, pseudophaeochromocytoma, phaeochromocytoma, catecholamines, adrenaline (epinephrine), noradrenaline (norepinephrine), 3-methoxytyramine, metanephrines, normetanephrine, metanephrine and dopamine, and hypertension. No language or data filters were applied. The Boolean operators “AND” and “OR” were used to combine search terms. Relevant studies were hand-searched within these references.

### Selection criteria

Studies reported catecholamines or metanephrines as primary outcome from CPAP treatment in patients with OSA in the absence of phaeochromocytoma, as confirmed by radiological investigations including computerised tomography or magnetic resonance imaging scans and metaiodobenzylguanidine or octreotide scans, as well as endocrine procedures such as a pentolinium suppression test and adrenal venous sampling.

### Risk of bias

The quality of the reports was evaluated using the risk of bias in non-randomized studies of interventions (ROBINS-I) tool that covers seven distinct domains: Two pre-intervention domains (bias due to confounding and selection of participants into the study), one domain at intervention (classification of interventions), and four post-intervention domains (deviations from intended interventions, missing data, measurement of outcomes and selection of the reported results.^12^ The risk of bias for each report was rated independently from low, moderate, serious to critical level by two authors and any discrepancies were resolved by reciprocal discussion.

### Statistical analysis

Meta-analysis of individual patient data was conducted using Bayesian approach to synthesise the evidence and estimate relative treatment effects for all procedures within the case studies. This technique was used for its ability to handle studies with small sample size, allowing the incorporation of prior information on model parameters and derivation of effect size from the posterior distribution for all studies based on Markov chain Monte Carlo simulations.^13^ When an unexplained heterogeneity occurred, a random-effects model with a common heterogeneity parameter was preferred to a fixed-effects model. Deviance information criteria were used to assess goodness-of-fit of the models. The analysis included non-informative priors for model parameters, and ran Markov chain Monte Carlo sampling for four chains, where first 1,00,000 posterior samples burn-in period were discarded and then another 1,00,000 posterior samples were saved in an interval of 10 in each chain.^14^ Convergence was attained based on visual inspection of time-series plots and using the Brooks-Gelman-Rubin test.^13^ The treatment effects were obtained from the posterior distributions of the Bayesian analysis and reported as a mean difference with 95% associated credible interval, which is a Bayesian analogy of the 95% confidence interval from traditional meta-analyses.^15^ The results were presented as forest plots, which display the mean difference for both the individual trials and also the pooled results. Because the reference ranges for the levels of catecholamines were variably reported between studies, percentage changes in post-CPAP treatment levels relative to pre-CPAP treatment levels were also presented. The analysis and graphics were performed and produced using the software R v3.6.2 and Just Another Gibbs Sampler (JAGS) which generates inferences.^13,16^

## Results

**Figure 1.**
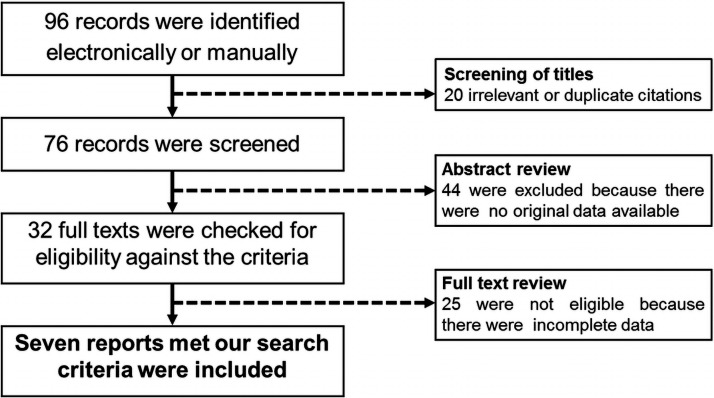
QUORUM flow chart of literature search.

The initial search yielded 96 potentially relevant records. Screening of titles identified 20 irrelevant or duplicate citations. After a review of abstracts, 44 articles were excluded because the original data were unavailable. Further detailed evaluation of full text found seven studies met our search criteria (Figure 1), comprising a total of 13 cases (seven men and six women).^17–23^ Nine cases had 24-hour urinary noradrenaline assessment before and after CPAP treatment (cases 1–8)^17–20^ or before and after a weight loss of 15 kg (12%) body weight in one patient (case 9).^20^ Of the remaining four cases, one had noradrenaline measured at baseline and normetanephrine/creatinine ratio after CPAP treatment (case 10),^20^ two had 24-hour urinary normetadrenaline measured (case 11 and case 12),^21,22^ and one had catecholamines measured but no details of the levels were available (case 13).^23^ The patients had mean age of 49.1 years (range: 36–62 years) and body mass index of 37.4 kg/m^2^ (range: 27–56 kg/m^2^). All patients were in the obese category (body mass index ≥30 kg/m^2^), except one patient who was overweight (case 3; body mass index of 27 kg/m^2^). Most patients had moderate to severe OSA, with CPAP treatment from a few weeks to several months (Table 1). Radiological investigations and endocrine dynamic function tests confirmed none of the patients had evidence of phaeochromocytoma (Table 2).

**Table 1.** Clinical characteristics and changes in blood pressure with CPAP treatment.

	Baseline characteristics						Blood pressure (mmHg)		
	Sex	Age (years)	BMI(kg/m^2^)	BP drugs (n)	AHI (events/hr)	OSA severity	Pre-CPAP	Post- CPAP	CPAP duration
1. Hoy et al., 2004^17^	F	62	56.0	1	>30	Severe	204/100	135/75	Not stated
2. Hoy et al., 2004^17^	F	52	41.0	3	15–30	Moderate	200/110	120/80	Not stated
3. Hoy et al., 2004^17^	M	42	27.0	3	>30	Severe	160/110	135/84	Not stated
4. Hoy et al., 2004^17^	M	48	35.0	3	15–30	Moderate	170/100	140/85	Not stated
5. Hoy et al., 2004^17^	F	38	36.0	3	15–30	Moderate	160/100	130/75	Not stated
6. Makino et al., 2006^18^	F	55	35.4	4	>30	Severe	247/140	160/100	2 weeks
7. Cheezum et al., 2010^19^	M	39	30.0	3	112	Severe	156/89	116/76	4 weeks
8. Kahal et al., 2013^20^	M	39	35.0	2	52	Severe	180/120	146/95	6 months
9. Kahal et al., 2013^20^	M	68	36.0	0	10	Mild^‡^	134/77*	--	--
10. Kahal et al., 2013^20^	M	51	42.0	4	40	Severe	174/133	--	2 years
11. Brainard et al., 2014^21^	M	36	--	2	--	Positive	154/104	118/82	7 weeks
12. Weeks et al., 2015^22^	F	56	43.8	0	--	Positive	166/100	--	7 weeks
13. Marmouch et al., 2021^23^	F	52	31.0	4	>30	Severe	200/120	130/80	4 weeks
All cases	7M: 6 F	49.1	37.4				177/109	133/83	

BMI, body mass index; BP, blood pressure; AHI, apnoea hypoapnoea index; OSA, obstructive sleep apnoea; CPAP, continuous positive airway pressure.

*This patient had mild OSA after weight loss of 15 kg (12% of body weight).

**Table 2.** Urinary screening test and radiological investigations.

	Investigations		
	24-hour urinary screening test	Adrenal CT	MIBG
1. Hoy et al., 2004^17^	Noradrenaline	Adenoma	Normal
2. Hoy et al., 2004^17^	Noradrenaline	Normal	Normal*
3. Hoy et al., 2004^17^	Noradrenaline	Normal	Normal*
4. Hoy et al., 2004^17^	Noradrenaline	Normal	Normal
5. Hoy et al., 2004^17^	Noradrenaline	Normal	Normal
6. Makino et al., 2006^18^	Noradrenaline	Normal	Normal
7. Cheezum et al., 2010^19^	Noradrenaline	Normal	Normal^†^
8. Kahal et al., 2013^20^	Noradrenaline	Normal	Normal
9. Kahal et al., 2013^20^	Noradrenaline	Normal	Normal
10. Kahal et al., 2013^20^	Noradrenaline^§^	--	--
11. Brainard et al., 2014^21^	Normetadrenaline	--	--
12. Weeks et al., 2015^22^	Normetadrenaline	Adenoma^¶^	--
13. Marmouch et al., 2021^23^	Noradrenaline	Normal	Normal

CT, computerised tomography; MIBG, metaiodobenzylguanidine.

*Both MIBG and octreotide tests were done; ^†^Only octreotide test was done. ^§^Only done at baseline; ^¶^Adrenalectomy showed adrenal cortical adenoma (no evidence of phaeochromocytoma)..

**Figure 2.**
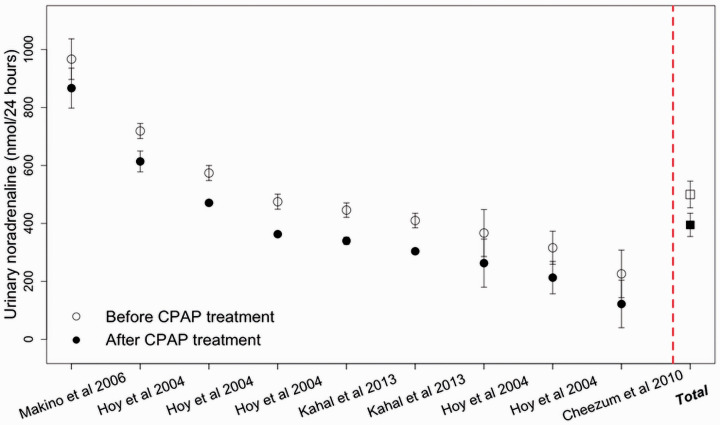
Noradrenaline levels before (○) and after CPAP treatment (●) for individual cases and for pooled results (□open square = before CPAP treatment, ▪ solid square = after CPAP treatment). Noradrenaline levels were reduced to within local reference ranges for all cases after CPAP treatment.

**Figure 3.**
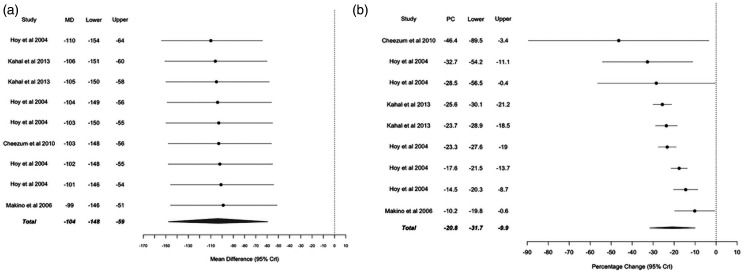
Mean difference (MD) in noradrenaline levels calculated as post-CPAP treatment adrenaline levels minus pre-CPAP treatment adrenaline levels (A), and percentage change (PC) in noradrenaline levels calculated as (post-CPAP treatment adrenaline levels minus pre-CPAP treatment adrenaline levels)/pre-CPAP treatment adrenaline levels (**B**) for individual cases (●) and for pooled results (♦).

Of the thirteen cases studied, CPAP treatment reduced the levels of catecholamines or metanephrines to within local reference ranges in each case except one, case 12 (92.3% of all cases).^22^ Analysis of individual cases and pooled data showed clear differences in noradrenaline levels before and after CPAP treatment (Figure 2). The absolute reduction of urinary noradrenaline levels after CPAP treatment was very similar among individual cases, regardless of the initial value. The overall mean (±SD) urinary noradrenaline before CPAP treatment was 499 ± 46 nmol/24-hours and after CPAP treatment was 395 ± 40 nmol/24-hours, *i.e.* a reduction of 104 nmol/24-hours (95% credible interval = 59 to 148 nmol/24-hours) (Figure 3(a)). Calculation of the percentage reduction of noradrenaline before and after CPAP treatment levels yielded an overall reduction of 21% (95% credible interval = 10 to 32%) (Figure 3(b)). For case 10, noradrenaline levels were raised by 30% above the upper reference limit at presentation (689 nmol/24 hours) which were normalised after CPAP treatment; although specific values for post-CPAP noradrenaline levels were not reported by the authors, post-CPAP normetanephrine/creatinine ratio for this patient was reported to be normal at 0.21 µmol/mmol (reference range <0.35 µmol/mmol).^20^ For the two patients (case 11 and case 12) where urinary normetadrenaline levels were reported, there was a reduction of 32.5% after CPAP treatment.^21,22^ Case 13 had noradrenaline levels at presentation of 3.5 times the upper limit of reference and were reduced to within reference limits after CPAP treatment.^23^

Mean systolic/diastolic blood pressure fell from 177/109 mmHg before CPAP to 133/83 mmHg after CPAP treatment, equating to a reduction of 31 mmHg (25.4%) in mean arterial pressure, from 131 mmHg to 100 mmHg, respectively. Because of the improvement in blood pressure, the number of antihypertensive medications that patients were taking before CPAP treatment was either unchanged or reduced in all cases after CPAP treatment (Table 1).

**Figure 4.**
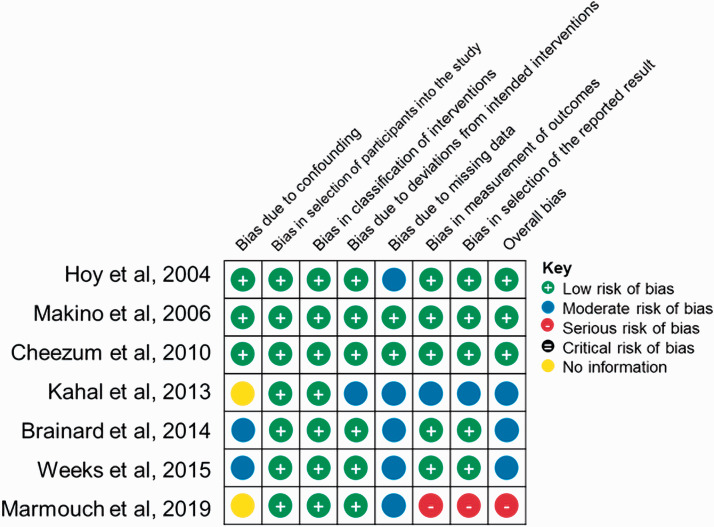
Risk of bias summary for included reports evaluated by the ROBINS-I tool.

The risk of bias evaluated by the ROBINS-I tool was found to be low in the majority of reports (Figure 4). The few issues identified were related to bias due to pre-intervention domains (lack of information on confounding factors such as co-morbidities in two studies) and post-intervention domains (incomplete data including the actual values of catecholamines) in one report.

## Discussion

The present study showed that the levels of catecholamines and metanephrines are reduced by CPAP treatment in those with OSA without evidence of phaeochromocytoma. These findings support the need to perform sleep studies for patients with hypertension who display features of OSA (snoring, witnessed apnoea, irregular breathing during sleep, restless sleeping, and chronic morning fatigue),^24^ before embarking on investigations for phaeochromocytoma.

In parallel to the reduction of catecholamines or metadrenalines, both systolic and diastolic blood pressure also fell to an acceptable target treatment range while the number of antihypertensive medications at presentation were either reduced or unchanged after CPAP treatment. These observations suggest that the removal of stress due to OSA, significantly diminished sympathetic overdrive^25,26^ in patients without evidence of phaeochromocytoma. Our findings are consistent with those observed in clinical studies of the effects of CPAP treatment on catecholamines and blood pressure in patients with OSA, but the reduction in blood pressure is greater in this study (31 mmHg) compared with that in clinical studies (<10 mmHg).^27,28^ There are a number factors that may contribute to this difference. In this study, phaeochromocytoma was excluded in all patients, therefore a good response to CPAP treatment would be expected. On the other hand, phaeochromocytoma may be present in clinical studies since this condition is not uncommon – estimated to be between one to six in a thousand individuals with hypertension^29–31^; such individuals would not respond so well to CPAP treatment due to autonomous catecholamine release. Other reasons may be due to the small numbers of subjects in case reports who may have been preferentially selected; the baseline blood pressure which appears to be high in reported cases; the duration of CPAP treatment and antihypertensive therapy; and compliance with CPAP may lead to a lack of treatment response, which may occur more frequently in clinical studies.

The levels of the other major stress hormone cortisol have also been shown to be elevated in patients with OSA. This is thought to be due to activation of the hypothalamic-pituitary-adrenal axis or the sympathetic nervous system by stress or disruptive sleep patterns in these individuals.^32^ Similar to the reduction in the levels of catecholamines demonstrated in this study of pseudophaeochromocytoma, abnormal screening tests such as 24-hour urinary free cortisol and the low-dose dexamethasone suppression test observed in untreated patients are also normalised by CPAP treatment in patients with pseudo-Cushing’s syndrome with coexisting OSA.^33^ Therefore, indiscriminate screening for phaeochromocytoma or Cushing’s syndrome without first excluding OSA would lead to a misdiagnosis, subjecting the patient to more invasive procedures and causing them unnecessary anxiety, as well as imposing additional costs to the healthcare system.

### Strengths and limitations

Although findings from case reports are often considered to provide weak scientific evidence, aggregation of results from case reports for systematic review and meta-analysis has been increasingly used.^34,35^ The advantage in the study of case reports is that a wealth of detailed individual data is available which can be synthesised and analysed.^36,37^ Studies have also shown that meta-analysis of case reports provides similar findings to those of clinical studies that comprise multiple participants.^38^ In addition, meta-analysis of individual patient data has also been shown to provide less bias and more reliable than meta-analysis of clinical trials.^39^ In the present study, we applied the ROBINS-I tool for assessing risk of bias^12^ and found the majority of the included reports to be at low risk of overall bias and with consistency between reports. These findings suggest good quality of the published data which enhances the overall certainty of evidence from individual reports on the reduction of catecholamine levels and blood pressure by CPAP. As with most meta-analyses, this study may suffer from potential bias from unpublished reports, especially those with negative results, leading to an over-estimation of the effect of CPAP treatment on blood pressure. The 44 articles excluded in this study were judged on the basis of their lack of data necessary for the purpose of our analysis (not due to negative results), therefore they would be unlikely to introduce bias to the findings of this study.

In conclusion, investigations of patients with suspected phaeochromocytoma, particularly obese individuals, should exclude OSA and treat this condition if present before performing screening tests to assess for catecholamine levels.
